# 

**Published:** 1874-01

**Authors:** 


Meeting of the State Medical Society.—The annual meeting of the Medical Society of the State of New York will be held in Albany February 3d, 4th and 5th. Several important questions are to be discussed at this meeting, among which will probably be the publication of the transactions, the propriety of changing the time of meeting, and the proposed change in the appointment of the nominating committee. The meeting will be an interesting one, and it is to be hoped that a large number of members and delegates will be present.

Reappointment.—Dr. E. C. W. O'Brien has been reappointed Health Physician by the new Board of Health. The appointment is one which will meet with approval, as the Doctor has shown during his terms of office that he is fully awake to the duties of his position. A better selection could not well be made.

———o———

We would call attention to the circular addressed to the Medical Profession at large in our Miscellaneous Department. The object is one in which all will sympathize, and we hope to see a lasting and appropriate monument erected to the memory of those faithful martyrs to the cause of humanity.

### Books and Pamphlets Received.

A Dictionary of Medical Science with the Accentuation and Etymology of the terms, and the French and other Synonyms. By Robley Dunglison M. D., LL. D. A New Edition Enlarged and thoroughly Revised. By Richard J. Dunglison, M. D. Philadelphia: Henry C. Lea, 1874. Buffalo: T. Butler & Son.

A Universal Formulary: Containing the Methods of Preparing Officinal and Other Medicines. By R. Eglesfeld Griffith, M. D. Third Edition Revised and Enlarged. By John M. Maisch, Phar. D. Philadelphia: Henry C. Lea, 1874. Buffalo: T. Butler & Son.

A Hand-Book of the Theory and Practice of Medicine. By Frederick T. Roberts, M. D, Bc. S, M. R. C. P. Philadelphia: Lindsay & Blakiston, 1874.

Sex in Education or a Fair Chance for the Girls. By Edward H Clarke, M. D. Boston: James R. Osgood & Co., 1873. Buffalo: Martin Taylor.

THE BUFFALO

**Medical and Surgical Journal**

PUBLISHED MONTHLY,

Containing Original Articles, Reports of Medical Societies and Hospitals, Editorial, Reviews, Correspondence, Army News, etc., etc; including the usual variety of Medical Periodical Publications. Specimen copies sent on application.

Address Buffalo Medical & Surgical Journal, Buffalo, N. Y.

TERMS OF SUBSCRIPTION, - - $3.00 PER YEAR, IN ADVANCE.

